# Disturbance of autophagy-lysosome signaling molecule expression in human gastric adenocarcinoma

**DOI:** 10.3892/ol.2013.1773

**Published:** 2013-12-24

**Authors:** SHAO-HUA WEI, WEI LI, YANG LIU, DE-KANG GAO, JUN PAN, CHUN-WEI GU, HAO-RONG WU

**Affiliations:** Department of General Surgery, The Second Affiliated Hospital, Soochow University, Suzhou, Jiangsu 215004, P.R. China

**Keywords:** human gastric carcinomas, autophagy, lysosome, Beclin 1, apoptosis

## Abstract

Autophagy is classified as type II programmed cell death and may participate in tumorigenesis. However, changes in autophagy-lysosome signaling and the relationship between the apoptotic cascade and gastric cancer cells have not been fully elucidated. The present study investigated the induction of autophagy in poorly differentiated human gastric adenocarcinoma. Immunoblotting revealed markedly induced autophagy in low grade differentiated gastric adenocarcinoma, indicated by elevation of microtubule-associated protein 1 light chain 3-I/II conversion and Beclin 1 in human gastric carcinomas. In addition, the diffuse (poorly differentiated) subtype showed significantly elevated Lamp2 and cathepsin B protein levels. Concomitantly, significant induction of anti-apoptotic events were indicated by changes in B-cell lymphoma 2 (Bcl-2) and X-linked inhibitor of apoptosis protein levels. Notably, confocal laser microscope data indicated co-expression of Bcl-2 and Beclin 1 in poorly differentiated human gastric adenocarcinoma. Results of this study indicate that the autophagy-lysosome signaling participates in poorly differentiated human gastric adenocarcinoma and there are intracellular links between autophagic signaling and the apoptotic cascade.

## Introduction

Gastric carcinoma is one of the most common causes of cancer-related mortality worldwide, and the prolonged survival rate of patients with unresectable or metastatic gastric carcinoma remains low (1–3). Determining the expression profiles of key molecules involved in the survival pathways holds a great deal of promise in improving the rate of diagnosis and prognosis of patients with gastric carcinoma. Epidemiological studies have demonstrated that diffuse (poorly differentiated) gastric adenocarcinomas present tumor aggressiveness, as well as a poor prognosis and response to chemotherapeutic interventions (4,5). Autophagy has recently attracted attention as a novel potential target in cancer treatment (6–8).

Autophagy is a homeostatic cellular process that recycles proteins and organelles using lysosomal machinery (9,10). Depending on the stimulus and cell type, autophagy is a double-edged sword, acting as a cell death mechanism whilst also aiding the prolonged survival of cancer cells in tumorigenesis (11,12). Several lines of evidence suggest that autophagy and apoptosis can coexist or occur sequentially. In contrast to the pro-survival role of autophagy, this pathway can culminate with caspase-independent programmed cell death (13,14). Several proteins are involved in the molecular mechanisms that result in apoptosis or autophagy. For example, cathepsin B activation is cell type-specific and plays a role in apoptosis via BH3 interacting-domain death agonist cleavage, release of cytochrome c and subsequent caspase activation (15). More recently, Chen *et al* (16) reported that decreased expression of Beclin 1 in gastric adenocarcinoma may be important in the acquisition of a metastatic phenotype, suggesting that decreased Beclin 1 expression is an independent biomarker for a poor prognosis in patients with gastric adenocarcinoma. Thus, inducing or impairing autophagy for therapeutic purposes requires an in-depth molecular knowledge of this process in a number of cancer cell types. However, the autophagy-lysosome process in gastric adenocarcinoma has not yet been elucidated. Understanding the context-specific role for autophagy in cancer and the mechanisms involved may be important to guide autophagy-based therapeutic intervention.

It is evident that gastric adenocarcinomas have distinct subtypes but the significance of these subtypes remains unclear. The present study aimed to investigate the changes in autophagic pathways, as well as the intracellular link between autophagic signaling and the apoptotic cascade in poorly differentiated human gastric adenocarcinomas.

## Materials and methods

### Patients and tissue specimens

Tissue samples from 20 patients with gastric cancer were obtained from the archives of the Second Affiliated Hospital, Soochow University (Suzhou, China), between November 2009 and December 2011. Tumor grades were defined in accordance with the criteria of the World Health Organization (2000) (17). The tumor-node-metastasis (TNM) stage of all gastric adenocarcinomas was assessed according to the criteria of the sixth edition of the TNM classification of the International Union Against Cancer (2002) (18). All 20 samples were solitary intramucosal gastric cancers of poorly differentiated types (TNMII, 14 cases; TNMIII, 6 cases). In addition, matched adjacent gastric mucosal tissues removed for radical gastrectomy were included as controls. The Institute Research Medical Ethics Committee of the Second Affiliated Hospital of Soochow University granted approval for this study. The patients provided written informed consent for their participation in this study.

### Immunoblotting

The tissue samples were homogenized in homogenizing buffer containing 50 mmol/l Tris-HCl (pH 7.4), 0.5% Triton X-100, 4 mmol/l ethylene glycol tetraacetic acid, 10 mmol/l EDTA, 30 mmol/l sodium pyrophosphate, 1 mmol/l Na_3_VO_4_, 50 mmol/l NaF, 100 nmol/l calyculin A, 50 μg/ml leupeptin, 25 μg/ml pepstatin A, 50 μg/ml trypsin inhibitor and 1 mmol/l dithiothreitol. The homogenates were centrifuged for 20 min at 15,000 × g to pellet the cellular debris and the protein concentration of the supernatant was determined using the Bradford method (Bio-Rad, Hercules, CA, USA). Equal volumes of samples were resolved by 10–12% SDS-PAGE and electrotransferred onto a polyvinylidene difluoride transfer membrane. This was probed overnight with the indicated primary antibody at 4°C, followed by incubation with the relevant horseradish peroxidase-conjugated secondary antibody for 1 h at room temperature. The primary antibodies used for immunoblotting included light chain 3 (LC3) rabbit polyclonal antibody (Medical and Biological Laboratories, Ltd., Nagoya, Japan), Beclin 1 (Cell Signaling Technology*,* Inc., Beverly, MA, USA), cathepsin B (mouse monoclonal antibody; Abcam, Cambridge, UK), lysosome-associated membrane protein 2 (Lamp2), B-cell lymphoma 2 (Bcl-2; Santa Cruz Biotechnology, Inc., CA, USA), Bcl-2 and nineteen-kilodalton interacting protein-37 (BNIP3) and β-actin (mouse monoclonal antibody; Sigma, St. Louis, MO, USA). Immunoreactive bands were detected by autoradiography with enhanced chemiluminescence (Amersham Biosciences, Little Chalfont, UK).

### Confocal scanning immunofluorescence microscopy

Immunolocalization and changes in LC3 and cathepsin B in human gastric adenocarcinoma were examined by confocal microscopy. Briefly, slices were prepared for fresh frozen coronal sectioning (15 μm thick). For immunohistochemical staining, slices were incubated with antibodies against LC3 (rabbit polyclonal antibody; Cell Signaling Technology, Inc.) and cathepsin B, or Beclin 1 (goat polyclonal antibody; Santa Cruz Biotechnology) and Bcl-2, overnight at 4°C. This was followed by immunofluorescence using a standard protocol from Perkin-Elmer (Waltham, MA, USA). Immunofluorescence was visualized using a Zeiss LSM 510 confocal microscope (Carl Zeiss AG, Oberkochen, Germany).

### Statistical analysis

The statistical analysis of experimental data was performed using a Student’s paired t-test (comparison of two groups). P<0.05 was considered to indicate a statistically significant difference. All data were expressed as the mean ± standard deviation.

## Results

### Changes in autophagy signaling in human gastric adenocarcinoma

**Figure 1:**
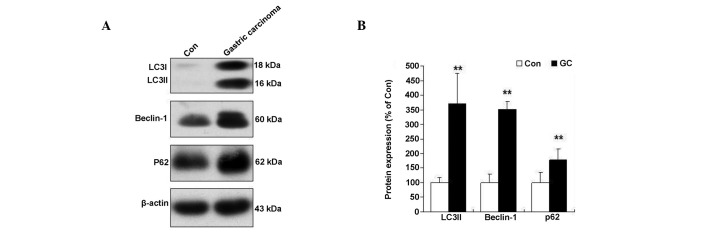
Changes in autophagy signaling molecules in human gastric adenocarcinoma. (A) A representative image showing protein expression of autophagic signaling molecules in human gastric adenocarcinoma using immunoblotting. Proteins were separated by SDS-PAGE and analyzed by immunoblotting with anti-Beclin 1, anti-P62 and anti-LC3 antibodies. (B) Quantitative analysis of protein levels was performed by densitometric analysis of the bands. Immunoblotting with an anti-actin antibody showed equal amounts of loaded protein in each lane. Data are expressed as percentages of control values (mean ± standard deviation; n=5). ^**^P<0.01, vs. control. Con, control; GC, gastric carcinoma; LC3, light chain 3.

The protein changes in autophagy signaling were monitored as a means of assessing autophagy progression in human gastric adenocarcinoma. The induction of an autophagy event was indicated by detection of membrane-bound LC3 phosphatidylethanolamine conjugate (LC3-II) by immunoblotting (19). The data showed LC3-I/II conversion in patients with low grade differentiated gastric adenocarcinoma (Fig. 1). The autophagy protein p62, a marker of autophagosome formation, was also monitored (20). A significant elevation of p62 was observed in patients with low grade differentiated gastric adenocarcinoma (Fig. 1). In addition, Beclin 1 is a critical component in the class III phosphatidylinositide 3 kinase complex that induces the formation of autophagosomes in mammalian systems (16). A significant increase of Beclin 1 was observed in patients with low grade differentiated gastric adenocarcinoma (Fig. 1).

### Expression of Lamp2 and cathepsin B protein levels in human gastric adenocarcinoma

**Figure 2:**
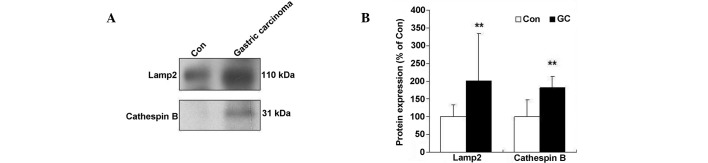
Protein expression of cathepsin B and Lamp2 in human gastric adenocarcinoma. (A) A representative image showing lysosome signaling in human gastric adenocarcinoma using immunoblotting. Proteins were separated by SDS-PAGE and analyzed by immunoblotting with anti-cathepsin B and anti-Lamp2 antibodies. (B) Quantitative analysis of protein levels was performed by densitometric analysis of the bands. Immunoblotting with an anti-actin antibody showed equal amounts of loaded protein in each lane. Data are expressed as percentages of control values (mean ± standard deviation; n=5). ^**^P<0.01, vs. control. Con, control; GC, gastric carcinoma; Lamp2, lysosome-associated protein 2.

It is becoming increasingly clear that the accumulation of autophagosomes alone cannot be used as an indicator of increased autophagy. Thus, measurement of lysosomal protein levels is essential for reflecting the function of the entire autophagy pathway (10). In the present study, the lysosomal protein expression was similar to the patterns observed for LC3-II formation, including the induction of Lamp2 and cathepsin B in patients with low grade differentiated gastric adenocarcinoma (Fig. 2). Lamp2 protein levels increased 2.0-fold in poorly differentiated human gastric carcinomas, compared with normal tissues. A significant decrease in the processing of the precursor forms of cathepsin B to their lower molecular weight mature forms (31 kDa) was observed in human gastric adenocarcinoma (Fig. 2). Thus, these data indicate that the lysosome process is involved in low grade differentiated gastric adenocarcinoma.

### Immunofluroscence of autophagy-lysosome signaling in human gastric adenocarcinoma

**Figure 3:**
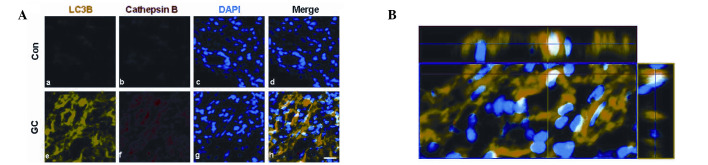
Immunofluroscence of autophagy-lysosome signaling molecules in human gastric adenocarcinoma. (A) Changes in the subcellular localization of cathepsin B (b and f; red fluorescence) and LC3 (a and e; green fluorescence) in human gastric adenocarcinoma. DAPI counterstaining indicates cell nuclei (blue). Representative images from one of three independent experiments are shown (scale bar, 20 μm). (B) The subcellular localization of LC3 and cathepsin B was demonstrated by XZ reconstructions. Con, control; GC, gastric carcinoma; LC3, light chain 3.

To further study autophagy-lysosome signaling in human gastric adenocarcinoma, immunocytochemical studies were performed using anti-LC3 and anti-cathepsin B antibodies. When human gastric adenocarcinomas were dual-labeled with antibodies, formation of LC3 and immunoreactivity against cathepsin B markedly increased compared with normal tissues (Fig. 3). As shown in Fig. 3B, the LC3 puncta largely colocalized with cathepsin B immunoreactivity in poorly differentiated human gastric carcinomas, indicating that gastric adenocarcinomas undergo both stages of the autophagy/lysosome process.

### Changes in anti-apoptotic signaling in human gastric adenocarcinoma

**Figure 4:**
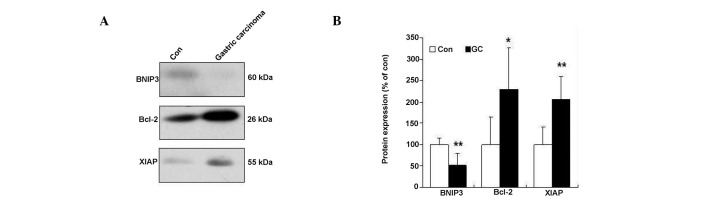
Changes in anti-apoptotic signaling molecule expression in human gastric adenocarcinoma. (A) A representative image showing anti-apoptotic signaling molecules in human gastric adenocarcinoma using immunoblotting. The proteins were stained with antibodies against BNIP3, Bcl-2 and XIAP. (B) Quantitative analysis of protein levels was performed by densitometric analysis of the bands. Immunoblotting with an anti-actin antibody showed equal amounts of loaded protein in each lane. Data are expressed as percentages of control values (mean ± standard deviation; n=5). ^*^P<0.05 and ^**^P<0.01, vs. control. Con, control; GC, gastric carcinoma; Bcl-2, B-cell lymphoma 2; BNIP3, Bcl-2 and nineteen-kilodalton interacting protein-37; XIAP, X-linked inhibitor of apoptosis.

To gain further insight into the changes in anti-apoptotic signaling in human gastric adenocarcinoma, the changes in BNIP3, Bcl-2 and X-linked inhibitor of apoptosis (XIAP) were determined by immunoblotting. The data demonstrated that BNIP3 levels significantly decreased in patients with low grade differentiated gastric adenocarcinoma. Consistent with this finding, similar results were obtained showing XIAP and Bcl-2 protein levels to increase in patients with low grade differentiated gastric adenocarcinoma (Fig. 4).

### Disturbance of Bcl-2/Beclin 1 is associated with cross-talk between the apoptotic cascade and autophagic signaling

**Figure 5:**
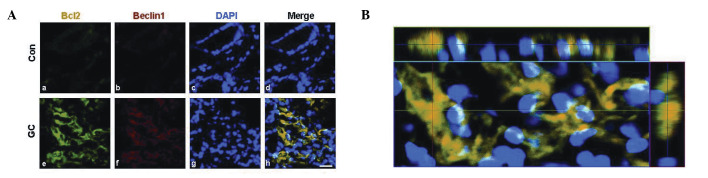
Immunohistochemical localization of Beclin 1 and Bcl-2 in human gastric adenocarcinoma. (A) Double immunostaining with anti-Bcl-2 (green fluorescence) and anti-Beclin 1 (red fluorescence) antibodies showed that (f) increased Beclin 1 expression was associated with (e) Bcl-2 activation in human gastric adenocarcinoma (scale bar, 20 μm). (B) The colocalization of Beclin 1 and Bcl-2 was demonstrated by XZ reconstructions. Con, control; GC, gastric carcinoma; Bcl-2, B-cell lymphoma 2.

The balance of Bcl-2/Beclin 1 signaling also plays a critical role during the cross-talk between the apoptotic cascade and autophagic signaling (21–23). It was reported that Beclin 1 expression was inversely correlated with tumor differentiation, nodal and distant metastasis and tumor relapse (24–26). To further study this result, immunocytochemical studies were performed using anti-Bcl-2 and Beclin 1 antibodies. When gastric adenocarcinoma cells were dual-labeled with these two antibodies, Bcl-2 immunoreactivity colocalized with Beclin 1 immunoreactivity in poorly differentiated human gastric adenocarcinoma (Fig. 5). Immunoreactivities against Bcl-2 and Beclin 1 markedly increased compared with those observed in normal tissue (Fig. 5).

## Discussion

The role of autophagy in oncogenesis and tumor survival is the subject of ongoing debate and remains unresolved (19). Results of the present study demonstrate that autophagic signaling participates in the pathological process of low grade differentiated gastric adenocarcinoma. In addition, Bcl-2/Beclin 1 signaling may be the intracellular link between autophagic signaling and the apoptotic cascade. Therefore, molecules involved in autophagic signaling in gastric adenocarcinoma may represent potential prognostic and therapeutic markers.

The biological significance and clinical impact of the variation in expression of autophagic markers in cancer appear to be associated with tumor types and tissue context (11,12). Therefore, it follows that identification of the specific autophagy profile in tissue specimens may offer important information for the diagnosis and tailored treatment of this cancer. The majority of gastric cancers are gland-forming adenocarcinomas (1). In the present study, the observed alternations in autophagy signaling were based on measurements of p62 and LC3 in human low grade differentiated gastric adenocarcinoma. The biochemical hallmark of autophagic initiation is the formation and subcellular redistribution of LC3-II (9,10). Significant elevations of LC3-II/I and p62 were observed in human gastric adenocarcinoma. Autophagy captures intracellular components in autophagosomes and delivers them to lysosomes, where they are degraded and recycled (9,10). In light of the key role of lysosome signaling in autophagy and apoptosis, the alternations in autophagy-lysosome progression were studied next, based on measurements of Lamp2 and cathepsin B levels in human gastric adenocarcinoma. Significant elevation of cathepsin B and Lamp2 levels in human gastric adenocarcinoma was found. Thus, induction of autophagy-lysosome signaling participates in the pathological processes of poorly differentiated human gastric adenocarcinoma.

There remains much controversy over the precise clinicopathological significance of autophagy signaling in gastric cancer (27). Recent evidence indicates that excessive autophagy may confer a tumor suppressive function and trigger a subsequent caspase-dependent apoptotic pathway (28). By contrast, Wojtkowiak *et al* (12) have shown that chronic autophagy occurs as a survival adaptation in mouse tumors. In the present study, protein analysis of human gastric adenocarcinoma revealed significant upregulation of autophagy regulatory proteins, LC3, cathepsin B and Lamp2. However, anti-apoptosis markers, particularly XIAP, were markedly increased in low grade differentiated gastric adenocarcinoma cells. Additionally, protein levels of the apoptosis inducer, BNIP3, decreased in the low grade differentiated gastric adenocarcinoma cells. Thus, data suggests that autophagy-lysosome signaling participates in poorly differentiated gastric adenocarcinoma in humans, and may be associated with poor prognosis in gastric carcinoma.

These observations bring into question whether the gastric adenocarcinoma-mediated apoptotic cascade is linked to autophagic signaling, and participates in the progression of human gastric adenocarcinoma. To evaluate the possible relationship between the apoptotic cascade status and autophagic signaling, immunoblotting was conducted to analyze changes in the autophagic protein, Beclin 1, which has long been identified as a Bcl-2-interacting partner (29). Results demonstrated that Beclin 1 is highly expressed in low grade differentiated gastric adenocarcinoma cells, compared with normal mucosal tissues. The dimeric mitochondrial protein encoded by the Beclin 1 gene is known to induce apoptosis (29). A significant elevation of survival protein Bcl-2 was observed in the same context, indicating a possible binding of Beclin 1 to the survival protein Bcl-2. Here, the apoptotic events are characterized by the activation of anti-apoptotic proteins, for example decreased BNIP3 and elevated XIAP in human gastric adenocarcinoma, which interact with apoptotic and/or anti-apoptotic molecules in human gastric adenocarcinoma.

Although variable loss expression of Beclin 1 has been observed in several types of human tumors (30), the relationship between Beclin 1 expression and gastric adenocarcinoma patient survival remains unclear. At present, elevation of Beclin 1 protein levels in human cancers (31) and decreases in Beclin 1 expression have been reported (32). Bcl-2 antiapoptotic proteins inhibit Beclin 1-dependent autophagy (21). With regard to gastric adenocarcinoma, double immunostaining analyses of the present study demonstrated colocalization of Beclin 1 and Bcl-2-positive immunoreactivity in human gastric adenocarcinoma. Consistent with this, Ahn *et al* (33) analyzed expression of Beclin 1 in a number of gastric adenocarcinomas. Weak or no immunoreactivity was detected in surface mucosal and mucosal glandular cells. In the context of the present study, we hypothesize that Bcl-2/Beclin 1 signaling may be a critical regulator of the biological effects of gastric adenocarcinoma and may be a key signaling element for the autophagic process, reflecting the prognostic result of patients. However, there are various other regulators of autophagic cell death that are involved in autophagic cell death in gastric carcinoma (34,35). Clearly, further studies are required to fully understand the potential function of Beclin 1 in human gastric carcinoma pathogenesis and to identify the signaling pathway involved in the tumorigenesis.

In summary, results of the present study indicate that autophagy-lysosome cascades may represent a low grade differentiated gastric adenocarcinoma feature of carcinoma cells. Furthermore, we hypothesize that increased Bcl-2/Beclin 1 signaling in human gastric adenocarcinoma may contribute to the interplay of apoptotic and autophagic events. The present study indicates that increased expression of autophagy-lysosome signaling molecules represents a new adverse independent prognostic factor in low grade differentiated gastric carcinoma. This insight may lead to an improved understanding of the role of autophagy in poorly differentiated human gastric adenocarcinoma. In addition, it may act as a valuable biomarker for the development of improved aggressive postsurgical adjuvant anticancer therapies.
